# Maintenance of NAD+ Homeostasis in Skeletal Muscle during Aging and Exercise

**DOI:** 10.3390/cells11040710

**Published:** 2022-02-17

**Authors:** Li Li Ji, Dongwook Yeo

**Affiliations:** 1 The Laboratory of Physiological Hygiene and Exercise Science, School of Kinesiology, University of Minnesota Twin Cities, Minneapolis, MN 55455, USA; 2 Department of Orthopedic Surgery, Mayo Clinic, Rochester, MN 55905, USA; yeo.dongwook@mayo.edu

**Keywords:** aging, exercise, NAD+, mitochondria, skeletal muscle, sirtuin

## Abstract

Nicotinamide adenine dinucleotide (NAD) is a versatile chemical compound serving as a coenzyme in metabolic pathways and as a substrate to support the enzymatic functions of sirtuins (SIRTs), poly (ADP-ribose) polymerase-1 (PARP-1), and cyclic ADP ribose hydrolase (CD38). Under normal physiological conditions, NAD+ consumption is matched by its synthesis primarily via the salvage pathway catalyzed by nicotinamide phosphoribosyltransferase (NAMPT). However, aging and muscular contraction enhance NAD+ utilization, whereas NAD+ replenishment is limited by cellular sources of NAD+ precursors and/or enzyme expression. This paper will briefly review NAD+ metabolic functions, its roles in regulating cell signaling, mechanisms of its degradation and biosynthesis, and major challenges to maintaining its cellular level in skeletal muscle. The effects of aging, physical exercise, and dietary supplementation on NAD+ homeostasis will be highlighted based on recent literature.

## 1. Introduction

Nicotinamide adenine dinucleotide (NAD+) is made of two covalently bound mononucleotides, a nicotinamide mononucleotide (NMN) and AMP. NAD+ plays a critical role in a wide range of cellular functions related to metabolism, signal transduction, and redox balance [1,2,3]. Discovered by Sir Arthur Harden more than a century ago as a chemical catalyst for fermentation and alcohol production [4], this classical coenzyme participates in numerous chemical reactions in the cell, yet its thorough biological role has not been revealed until the past several decades [1,5,6]. Originally, NAD+ was considered mainly a reducing power, accepting hydrogen ions from various energy substrates while being reduced to NADH, thus supporting oxidative and reductive chemical reactions in the intermediate metabolism [7,8]. This reaction is reversible when NADH is converted back to NAD+, therefore total NAD+ and NADH pool would be maintained constant [3]. However, when NAD+ is used in non-redox reactions, such as during signal transduction, its role has widely expanded, and its levels are depleted.

The recognition of NAD+ as a multifunctional signaling molecule has been largely driven by the research of aging and nutrition. In the yeast and invertebrates, NAD+ supplementation increases life span and defies age-related functional deteriorations [9,10]. In mammals and humans, dietary supplementation of NAD+ precursors, such as nicotinic acid (NA), nicotinamide mononucleotide (NMN) and nicotinamide ribose (NR) provides clear amelioration in age-related mitochondrial deterioration, metabolic dysfunction, insulin resistance, neurological disorder, and exercise capacity [2]. The studies of sirtuins (SIRTs), a family of NAD+-dependent protein deacetylase, have provided a mechanistic link between NAD+ homeostasis and cell functionality. Increased protein acetylation underlies numerous age-related muscle disorders, such as mitochondrial deterioration (due to PGC-1α and FoxO3a hyperacetylation), reduced antioxidant defense (mainly due to MnSOD acetylation), and increased inflammation (due to acetylation of p65) [11]. Deacetylation (as well as the removal of succinyl, malonyl, glutamyl, and palmitoyl moieties) of proteins including transcription factors requires higher SIRT activity, but ironically, SIRT1 activity in skeletal muscle is decreased with aging despite an increase in SIRT1 protein expression [3,12]. As a mandatory substrate for SIRTs, decreased NAD+ availability limits SIRT1 ability for deacetylation, whereas maintenance of NAD+ pool ensures SIRT functions in promoting longevity in yeast, certain invertebrate species, and mice, and enhancing disease resistance in mammals and human [13,14,15].

As a carrier of ADP-ribose, NAD+ also activates poly (ADP-ribose) polymerase (PARP) to transfer multiple ADP-ribose to generate long-chair poly ADP-ribose (pADPr) to repair DNA backbone and other proteins [2]. In skeletal muscles of old mice, PARP-1 was upregulated while pADPr levels were elevated [12]. Interestingly, PARP-1 inhibition improved mitochondrial function due to SIRT1 activation, presumably by increased NAD+ availability [16]. In addition to SIRTs and PARP as the main NAD+ consumers, NAD+ can be directly hydrolyzed by CD38 and CD157, a group of NADases that use NAD+ to generate pADPr and cyclic ADP-ribose (only 3%), the latter serving as a Ca^2+^ ion modulator [1]. Aging increases CD38 protein level and NADase activity in skeletal muscle [3,12], whereas inhibition of CD38 or CD38 gene knockout was shown to rescue intracellular NAD+ and preserve SIRT activity [17]. During aging, each of the three major NAD+-consuming pathways is activated and competes for NAD+ as a substrate.

**Figure 1:**
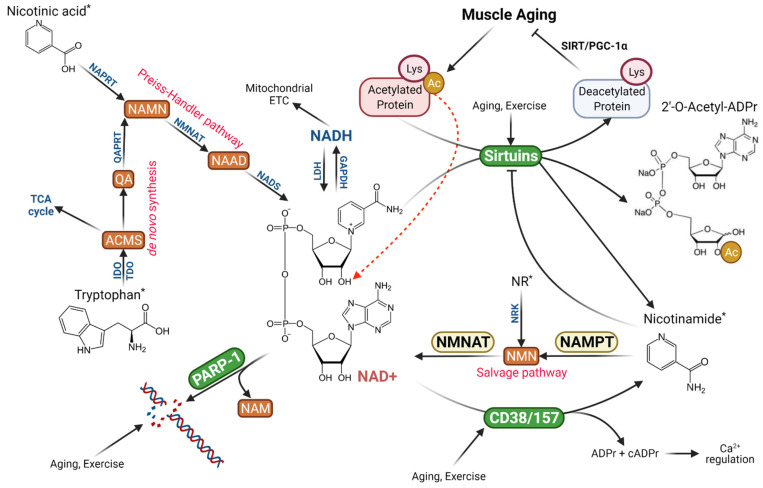
Schematic illustration of maintaining NAD+ levels in skeletal muscle during aging and exercise. Arrows with blunt ends indicate inhibition. Compounds with an * denote dietary sources. See abbreviation list for details of enzyme and compound nomenclatures. Figure created with BioRender.com.

Theoretically, increased NAD+ consumption can be replenished by elevated NAD+ synthesis in the cell. The Preiss–Handler pathway converts dietary NA to nicotinic acid mononucleotide (NAMN), then nicotinic acid adenine dinucleotide (NAAD), and finally NAD+, whereas the de novo NAD+ biosynthesis from dietary tryptophan requires multiples steps with limited utility [2]. A more important pathway relied on by organisms is the NAD+ salvage pathway controlled by nicotinamide phosphoribosyltransferase (NAMPT), the rate-limiting enzyme converting NAM cleaved by SIRTs, CD38 and PARP-1 to NMN, and eventually NAD+ (Figure 1). This pathway not only recycles NAM to maintain intracellular NAD+ levels but also relieves NAM inhibition on SIRTs [2]. There is evidence that the decline of NAMPT activity at old age reduces NAM recycling and NAD+ salvage, resulting in muscle degeneration [18]. On the other hand, dietary supplementation of NAM or NR can boost intracellular and mitochondrial NAD+ levels, improve metabolic function and even increase longevity in aged mice [3].

The current review will highlight recent literature in NAD+ homeostasis with respect to its primary cellular functions, degradation, and biosynthetic pathways in the skeletal muscle. The effect of aging and muscle contraction on the regulatory mechanisms will be the focal points of this review because of their relevance to sarcopenia and exercise adaptation.

## 2. NAD+ Functioning as a Coenzyme

NAD+ has two major biological roles, serving as the reducing equivalent for metabolism and as a signaling molecule. For a long time, the role of NAD+ has been defined as the electron carrier between metabolic substrates and the mitochondrion [4]. Hydrogen ions are transferred from glyceraldehyde 3-phosphate to 3-phosphoglyceryl phosphate via glyceraldehyde phosphate dehydrogenase (GAPDH), the only site to produce NADH in glycolysis. In the mitochondria, NADH is produced in several enzymatic steps catalyzed by pyruvate dehydrogenase (PDH), isocitrate dehydrogenase (ICDH), α-ketoglutarate dehydrogenase (α-KGDH), and malate dehydrogenase (MDH) in the TCA cycle, 3-hydroxylacyl coenzyme-A dehydrogenase (HADH) in fatty acid β-oxidation, and glutamate dehydrogenase (GDH) and branched-chain α-ketoacid dehydrogenase (BCKADH) in amino acid metabolism. NADH formed in the TCA cycle is directly delivered to NADH dehydrogenase of the Complex I of the electron transport chain (ETC) to produce ATP, whereas cytosolic NADH needs to be transported into the mitochondria by two mitochondrial inner membrane shuttle systems, the glycerol-3-phosphate shuttle or the malate-aspartate shuttle. In the former case, glycerol-3 phosphate dehydrogenase (GPDH) couples NADH oxidation with FAD moiety of the ETC Complex II, whereas in the latter case, malate-generated NADH are delivered to the ETC at Complex I [19].

Whether or not oxygen supply is available to receive electrons from ETC to form water, simultaneously generating ATP, is crucial to keeping electron flow from energy substrates to mitochondria. If O_2_ supply becomes limited, NADH accumulates, not only shutting down inner member NADH shuttles mentioned above but also inhibiting the very enzymes sensitive to rising NADH/NAD ratio, such as PDH, ICDH, and α-KGDH [19]. This inhibition not only decreases acetyl-CoA entry into the TCA cycle but also limits pyruvate oxidation causing NADH accumulation in the cytosol. Lactate dehydrogenase (LDH) utilizes NADH to reduce pyruvate to lactic acid, providing a metabolic outlet of electrons under anaerobic conditions and thus, a huge advantage for muscle to utilize carbohydrate (mainly glycogen) with a low oxygen supply. Meanwhile, NAD+ is regenerated by LDH to supply GAPDH to continue the enzymatic reaction to maintain glycolytic flux.

In the pathways of biosynthesis, such as the fatty acid synthase complex (FAS) to generate palmitate from malonyl coenzyme-A, reducing powers are needed to provide hydrogen ions to each of the elongation steps in the form of NADPH instead of NADH. NADPH also provides reducing power for the biosynthesis of other important molecules, such as cholesterol and DNA. NADPH plays a crucial role in the maintenance of cellular redox balance, wherein NADPH donates a pair of electrons to glutathione (GSH) and thioredoxin (TRX), via glutathione reductase (GR) and thioredoxin reductase (TR), respectively, to keep them in the reduced state. GSH and TRX serve to reduce α-tocopherol (vitamin E), ascorbic acid (vitamin C), and α-lipoic acid to neutralize reactive oxygen species (ROS) and maintain cellular antioxidant defense. During the above reactions, NADPH is oxidized to NADP+, whereas NADPH is regenerated by glucose 6-phosphate dehydrogenase (G6PDH) and 6-phosphogluconate dehydrogenase as part of the pentose shunt. An isozyme of ICDH in the mitochondria and peroxisome and malic enzyme can also generate NADPH [19].

In resting skeletal muscle, NAD+ concentration is estimated to be 1.5–1.9 mmol/kg dry wt (values multiple by ~4.3 per wet wt), among which 3.15 mmol/kg wet wt is located in the mitochondrial [20]. The NAD+/NADH ratio in resting skeletal muscle is estimated to be ~540 in the cytosol compared with ~6.3 in the mitochondria [20]. It is noteworthy that, in the cell, NAD+ is much more abundant than NADH, such that NAD+/NADH is kept high; in contrast, NADPH instead of NADP+ is the predominate form in the cell [1,2]. This profile has two important implications: (1) any fluctuation of total NAD+ level will minimally affect the NADH/NAD+ ratio, thus minimizing the effect on various enzymatic steps discussed above; and (2) any small change in NADH level will result in a large change in the NADH/NAD+ ratio, sensitive to many metabolic enzymes. For example, when oxygen is limiting and electron flow from NADH to ETC decreases, the NADH/NAD+ ratio rises and inhibits PDH, ICDH, and α-KGDH, thus slowing down flux through the TCA cycle.

Perturbation of intracellular NADH/NAD+ (NADPH/NADP+) not only affects various enzymes and pathways in metabolic functions but also intracellular redox status. Together with the glutathione (GSH)/glutathione disulfide (GSSG) ratio, NADH/NAD+ can modulate cellular production of ROS as well as antioxidant defense capacity, thereby determining whether the cell is subjected to oxidative stress or reductive stress [21]. It is generally believed that cellular NAD+ level increases in response to lower energy states, such as starvation, glucose deprivation, caloric restriction, and physical exercise. In contrast, NAD+ concentration decreases during energy abundance, such as feeding on a high-fat diet, and aging [2]. Hyperglycemia, a condition of excessive utilization of blood glucose as a fuel, such as with diabetes mellitus, overproduction of NADH in the cytosol coupled with impaired NADH oxidation in the mitochondria can lead to elevated NADH/NAD+ ratio and reductive stress [22]. An excess of NADH is known to impose stress on ETC, leading to increased electron leakage and superoxide anion production. The cell may respond by activation of the Nrf2 pathway to increase GSH levels and exacerbate reductive stress [21]. In other pathological conditions, such as diabetic cardiomyopathy and myocardial ischemia reperfusion injury, NADH/NAD+ ratio increases partly due to the activation of the polyol pathway (also known as the sorbitol-aldose reductase pathway) [21,23].

Compared to NADH/NAD+ ratio, the NADPH/NADP+ ratio is even more important in regulating cellular redox status, mainly because of its crucial role in GSH and TRX synthesis and redox cycle [24,25]. Protection against oxidative stress largely relies on the reductive power of NAPDH, whose levels are mostly determined by G6PD. It has been shown that increased NADPH production in transgenic mice overexpressing G6PDH lowered muscle oxidative damage, decreased aging-associated functional decline, and extended median lifespan [26].

## 3. NAD+ as a Signaling Molecule

During the past two decades, evidence emerged that NAD+ is an important signaling molecule and serves multiple cellular functions previously unknown or not entirely defined. The first and perhaps the best-known function of NAD+ is to serve as a mandatory substrate for SIRTs, the enzymes with either mono-ADP-ribosyltransferase or deacylase activity [13]. The latter has the ability to remove acetyl, succinyl, malonyl, myristoyl, or palmitoyl moiety from proteins, making it a versatile and critical enzyme involved in metabolic control, gene regulation, antioxidant, anti-inflammation and anti-aging, and other vital cellular functions [15]. There are seven SIRTs in the cytosol, mitochondria, and nucleus, but all of them catalyze a reaction to transfer the acetyl moiety of the target protein to ADP-ribose of the cleaved NAD+, resulting in a free NAM and O-acetyl-ADP-ribose [2] (Figure 1). Clearly, the SIRT-catalyzed reactions in the cell, regardless of what function it is, consume NAD+ molecules which are distinguished from converting NAD+ to NADH and will result in net reductions of NAD+ concentration if not properly replaced. Furthermore, the reaction releases NAM molecule, a strong inhibitor of SIRTs [27]. Thus, it has been shown that upregulation of CD38, a NAD+-hydrolyzing enzyme, during aging can reduce SIRT1 activity despite relatively stable SIRT1 protein content in the cell [12]. This may explain some of the age-related declines in metabolic functions, including but not limited to mitochondrial deterioration [28]. The effect of aging on NAD+ homeostasis in muscle will be discussed in later sections.

The second function of NAD+ relates to its ability to donate ADP-ribose connected as a poly-ADP-ribose chain, the primary DNA backbone structure in living organisms, and to histone and other proteins [2]. This reaction fulfills an important role in DNA repair at the damaging site and maintains proper genomic integrity [29]. The enzyme that catalyzes this reaction is PARP. There are several forms of PARP isozymes, but PARP-1 has been recognized as the most important one in mammals [5]. Sudden oxidative stress, such as the ionizing radiation during cancer chemotherapy, can cause a dramatic reduction of NAD+ levels due to the large consumption of NAD+ for DNA repair. In the muscle, 80% of NAD+ is thought to be consumed by PARP-1 to repair DNA damage [29]. Thus, drugs that inhibit PARP can be used to facilitate the therapeutic regimen for cancer treatment due to the prevention of DNA repair [30]. Because of the dual roles of NAD+ to provide substrate for both SIRTs and PARP, there is a competition between the two enzymes for the same cellular NAD+ pool. This competition is especially evident when cells are under oxidative challenges, such as heavy exercise, caloric restriction, antioxidant depletion, and aging [9,10,18,28].

NAD+ is also the only source of a signaling molecule called cyclic-ADP-ribose (cADPr), which is a second messenger in regulating Ca^2+^ release from intracellular storage via activation of the ryanodine channel [31]. Although the precise mechanism is not entirely clear, it may have significant implications in skeletal muscle, the contraction of which heavily depends on Ca^2+^ dynamics. The generation of cADPr requires the enzyme cyclic ADP ribose hydrolase (also known as cluster of differentiation 38 or CD38) to catalyze the hydrolysis NAD+ to produce both ADP-ribose (97%) and cADPr (3%). In muscle cells, CD38 functions as a NADase and consumes a large amount of NAD+, and interestingly, aging has been shown to upregulate CD38 [17,32]. Age-related upregulation of CD38 is thought to be the main reason for a decline of NAD+ levels in the cell [33,34]. Prevention of CD38 upregulation with inhibitor 78c, or using CD38 gene knockout, has been shown to prevent age-related NAD+ decline [17,32]. In contrast, an overexpression of CD38 is associated with exaggerated NAD+ reduction and mitochondrial dysfunction [35].

## 4. NAD+ Synthesis

### 4.1. Major Pathways for NAD+ Replenishment

Several pathways function to replenish cellular NAD+ levels and support a normal NAD+ turnover, but their utility and importance are different. The de novo NAD+ synthesis, or the so-called Kynurenine pathway, requires tryptophan as the precursor and several enzymatic steps to produce 2-amino-3 carboxymuconate semialdehyde (ACMS) and then quinolinic acid (QA). QA is converted to NAMN by the enzyme QA phosphoribosyltransferase (QAPRT) and joins the Preiss–Handler pathway. This pathway is conserved and operational in yeast, invertebrates, and humans, with some variations of the enzymes involved [1], but is limited by the activity of TDO and IDO and requires additional six steps to synthesize NAD+ [2]. Furthermore, under normal conditions, ACMS is decarboxylated and metabolized in the TCA cycle and does not lead to a meaningful replenishment of cellular NAD levels (Figure 1) [2]. A second pathway utilizes a major form of niacin (vitamin B3) NA to produce NAD+ via the Preiss–Handler pathway. NA is first converted to NAMN, which is then converted to NA adenine dinucleotide (NAAD) by NAMN transferase (NMNAT). The importance of NMNAT is highlighted by the fact that three forms of isoenzymes are localized in critical components of the cell, the nucleus (NMNAT1), cytosol (NMNAT2), and mitochondria (NMNAT3). A final step completes NAD+ formation from NAAD by NAD+ synthase (NADS) [2].

The most important pathway for NAD+ biosynthesis in the cell is the NAD+ salvage pathway, which utilizes NAM, the common product of the three NAD+-consuming pathways, namely SIRTs, PARP-1, and CD38, and the one that most mammals cannot synthesize de novo, as the precursor [2]. The first step of the pathway is catalyzed by NAMPT, the rate-limiting enzyme, to produce NMN. NMN is then converted to NAD+ by NMNAT (1–3) in various cellular compartments (Figure 1). The NAD+ salvage pathway regenerates NAD+ to maintain its cellular pool, at the same time removing NAM, which can bind with a conserved DNA sequence on SIRTs, PARPs, and CD38/CD157, and negatively regulate their catalytic activity [27]. Thus, NAD+ salvage ensures a stable cellular NAD+ pool to support these pathways stated above.

### 4.2. Efficacy of NAD+ Precursor Supplementation

Under normal condition, dietary source of niacin (NA, NAM) at a daily dose of 20 mg seems to sustain a healthy NAD+ turnover; however, higher NAD+ levels have been shown to ameliorate a number of cardiovascular, metabolic, and neurodegenerative disorders, and aging [36]. Therefore, there has been increasing interest in both research and clinical practices to boost NAD+ synthesis in the cell. So far, dietary supplementation of NAD+ precursors has been proven to have considerable efficacy, although exercise has gained increasing recognition as an effective regimen [37]. Besides tryptophan, four NAD+ precursors are generally accepted as potential dietary supplements, namely, NA, NAM, NMN, and NR [36].

Since the NAD+ salvage pathway constitutes a major route of NAD+ biosynthesis in vivo, NMN was first recognized as a potential source to mitigate NAD+ deficiency in mice due to SIRT1 overexpression, because NMN can be converted to NAD+ in a single step by NMNAT [38]. The effect of NMN supplementation on restoring NAD+ levels was later confirmed in wild-type mice [39], and to reverse aging effects at the cellular and organismal levels [28]. Theoretically, direct supplementation of NAM can be used to raise NMN levels due to the ability of NAMPT to convert NAM to NMN [2]. However, several pathways in the cell consuming NAD+, such as SIRTs, PARP-1, and CD38 generate NAM, which poses negative feedback to these enzymes due to the binding of NAM to a conserved DNA moiety as previously mentioned, thus the efficacy of NAM supplementation in vivo is somewhat doubtful. Another caveat is that NAMPT expression and activity are affected by aging, making the utility of recycling NAM in muscle uncertain [12]. Nevertheless, NAM supplementation was demonstrated to improve glucose homeostasis, reduce the acetylation level of Sirt1 targets, and increase NMNAT1 expression but without significant elevation of NAD+ levels in mice fed a high-fat diet [40].

Compared to L-tryptophan, NA, and NAM, dietary presence of NR is at a negligible level [41]. This dietary profile of NR makes it a potential supplement to counter NAD+ deficiency and protect against aging [9,42]. Importantly, NR can be converted to NMN by NR kinase (NRK), a conserved enzyme in humans [43]. Oral ingestion of NR has displayed excellent safety and bioavailability in humans, as shown by multiple pre-clinical trials and clinical trials [44]. Airhart et al. [45] reported in a pharmacokinetic study that oral supplementation of NR at 1000 mg/day for multiple days was well tolerated in human subjects and that NR increased blood NAD+ concentration by 100% compared with baseline levels. Recent literature confirmed that NR supplementation has some distinguished advantages as a dietary supplement (see Mehmel et al., 2020 for a detailed review). For example, Remie et al. [46] showed that NR supplementation of 1000 mg/day for 6 weeks increased muscle NAD+ metabolites, acetylcarnitine (as a fatty acids metabolism marker), and fat-free mass in overweight or obese human subjects. In aged human subjects, NR supplementation increased NAD+ metabolome in the skeletal muscle, suppressed circulating pro-inflammatory cytokine levels, and ameliorated functional deficits and muscle mass [47]. When NMNAT was knocked out in mice, supplementation of NR failed to protect muscle deterioration with aging, demonstrating the crucial role of NMNAT in NAD+ salvage. Recently, Yaku et al. [48] demonstrated in an elegantly executed study that in C57BL/6N mice, oral gavage of NR increased NAD+ levels in several tissues via two distinct pathways. In the early phase, NR was directly absorbed and boosted NAD+ generation through the salvage pathway. During the second phase, NR was first hydrolyzed to NAM by bone marrow stromal cell antigen 1 (BST1) and was then metabolized by the gut microbiota to NA, generating NAD+ through the Preiss–Handler pathway. Several recent review articles summarized and compared the bioavailability, pharmacokinetic, and pharmacological properties of NA, NAM, NMN, and NR [36,49,50]. Radenkovic et al. [49] conducted a systematic review of clinal trials using meta-analysis (with 36 studies fit into the inclusion criteria) and revealed some interesting trends. During the 1960–1990s, the majority of the studies employed direct NAD+ or NADH oral supplementation. Since the 1990s, more clinical trials were conducted using NAD+ precursors, such as NAM and NA. Entering the 2010s, a clear majority of published data was based on NR supplementation. Out of the 36 trials, 17 revealed positive effects of supplementation, treating a wide variety of diseases and disorders, whereas only 2 were adequately powered. Increased NAD+ levels in blood or tissues were reported in 11/36 studies, with variable sizes of effects. Noticeably, the authors included several studies with exercise interventions and emphasized its benefits. Trammell et al. [50] compared the efficacy of various NAD+ precursors supplementation in muscle and offered some conclusive remarks: (1) NR has the greatest oral bioavailability among the four precursors followed by NAM, which in turn is better than NA. (2) NR and NA supplementation can increase NAD+ content in muscle, but NMN cannot. (3) NR exhibits a greater ability to increase SIRT1 and SIRT3 activity (via measurement of ADP-ribose accumulation) than NAM due to their NAD+ salvage potential. (4) NR supplementation shows a specific advantage in promoting stem cell regeneration and improving resistance to chemotherapy-induced neuropathological disorders. A recent review by Khaidizar et al. [51] also reviewed the efficacy of NAD+ and NAD+ precursor supplementation and highlighted the benefits of NR over other compounds in ameliorating aging and age-related diseases. The comparisons among various NAD+ precursors may be complicated by the recent finding that gut microbiota is capable of converting NAM to NA and utilizing multiple pathways to synthesize NAD+ [48,52].

## 5. Impact of NAD+ Deficiency on Muscle Function

Skeletal muscle is a metabolically active tissue. It sustains most of glucose utilization and turnover, ATP production for physical work, and represents the reservoir of body protein source. Most of these functions require a constant supply of NAD+ as the acceptor of electron transfer. The operation of mitochondrial ETC ensures that NADH can be converted back to NAD+ and that cytosolic NAD+ is maintained stable. However, under certain conditions, this balance can be disturbed, resulting in a net deficit of NAD+ and loss of homeostasis.

### 5.1. NAD+ and Cell Signaling

SIRTs as one of the major NAD+ consumers play an important role in maintaining proper muscle functions. Since a significant amount of cellular proteins (including enzymes and transcription factors) are in acetylated state and as such, dysfunctional, SIRTs transfer the acetyl (as well as succinyl, malonyl, glutaryl, and fatty acyl) moieties to the ADP-ribose of NAD+, so that their proper functions can be restored [18,53]. For example, Sirt1 deacetylates PGC-1α and activates the Sirt1/PGC-1α axis for mitochondrial biogenesis and homeostasis [54]. Enhanced acetylation of the TCA cycle enzymes, such as CS and ICDH, can render the mitochondrion to decreased flux and ATP generation [11,55,56,57]. Located in the mitochondrion, Sirt3 is the main deacetylase that relies on the mitochondrial NAD+ pool. Sirt3 deacetylates mitochondrial superoxide dismutase (SOD2) to maintain its catalytic activity [12,58,59]. Decreased SOD2 due to NAD+ deficiency reduces mitochondrial ability to remove superoxide radicals and subject the organelle to oxidative stress [13,60]. Furthermore, deacetylation of FoxO3a is an important step in activating this crucial nuclear factor for antioxidant control, autophagy, and aging [54]. Inactivation of FoxO3a may downregulate NAMPT, the key enzyme for NAD+ salvage, further reducing NAD+ levels. Moreover, skeletal muscle regularly generates high force, which could subject muscle fibers to injury and subsequent inflammation. NFκB activation is a mandatory step in the inflammatory response, during which the p65 subunit of the NFκB complex translocates into the nucleus. Acetylation increases the binding activity of p65, whereas Sirt1 deacetylates p65, thus inhibiting its translocation [61,62]. If the muscle is deficient of NAD+, this anti-inflammatory effect is severely attenuated. Since there are seven SIRTs in the cell, with Sirt1, 6, and 7 mainly in the nucleus, Sirt2 in the cytosol, and Sirt3, 4, and 5 in the mitochondria, it is interesting to note that different compartments of the cell maintain a different concentration of NAD+, which in turn regulates various SIRT fractions differently [63]. In the muscle, Sirt1 protein content is relatively stable, but its activity varies depending on NAD+ local concentration [15,64,65]. During starvation, adrenergic receptor binding, and protein kinase A activation can phosphorylate Sirt1, which decreases the Km of Sirt1 for NAD+ and sensitizes the enzyme at lower NAD+ concentration [66]. Thus, a small reduction of NAD+ concentration would not seriously affect Sirt1 function. Sirt3 is the primary controller of mitochondrial metabolic flux through deacetylation and activation of enzymes in the TCA cycle, the urea cycle, and oxidative phosphorylation [67,68,69,70,71].

To separate the effects of NAD+ deficiency from that of aging, a common experimental model to study the impact of NAD+ is to deplete the NAD+ pool by inhibiting the salvage pathway. Frederick et al. [18] developed a NAMPT knockout (mNKO) mouse model and demonstrated that at 3 months of age, NAD+ content was reduced to <15% of normal concentration, confirming the essential role of the NAD+ salvage pathway. ATP production in these animals was reduced by >60%, associated with a decline of the mitochondria isolated from the mNKO mouse muscle to utilize pyruvate and palmitoyl carnitine for oxidative phosphorylation. NAD+ deficiency did not result in loss of muscle weight, fiber twitch-force generation, or endurance running capacity. However, at 7 months of age, the mMKO mice demonstrated decreased lean body mass and running performance. These physiological deficits appeared related to fiber necrosis under the plasma membrane. Perhaps the most striking discovery of the study was that NAD+ deficit due to NAMPT KO caused the alteration of 267 transcription factors related to muscle inflammation, metabolic function, ubiquitin proteolysis, and senescence [18]. These observations support a scenario that at young age, cellular NAD+ level is maintained in abundance and well above a threshold below which muscles develop symptoms of metabolic disorders [72]. Interestingly, glycolytic flux generated by GAPDH was not decreased and even elevated to compensate for declined mitochondrial ATP production.

Skeletal muscle periodically faces oxidative stress due to its high contractility that generates ROS. Oxidative modification of DNA results in oxidized nucleic acids, such as 8-hydroxy-dG [25]. Research shows that repair of DNA damage with the enzymatic reaction of PARP-1 is a major reason for NAD+ decline, accounting for 80% of the reduction [29]. PARP-1 transfers multiple ADP-ribose of NAD+ molecule to DNA and protein acceptors, forming pADPr chains, the backbone of DNA. In many cell types, PARP-1 levels are inversely related to NAD+ levels. It was shown that exposure of C2C12 cells to H_2_O_2_ can activate PARP-1 and a subsequent reduction of NAD+ concentration. This in turn, decreases SIRT1 activity, even though SIRT1 protein content is unchanged [16]. It is noteworthy that NAD+ is highly compartmentalized in the skeletal muscle and that ~95% of the NADH is localized in the mitochondria [53]. Because both PARP-1 and SIRTs (mainly Sirt1) are located in the nucleus, they share the same pool of NAD+. Thus, activation of PARP-1 under oxidative stress can drastically reduce NAD+ concentration in the nucleus and essentially outcompete SIRTs [73,74]. Therefore, inhibition of PARP-1 has been shown to conserve NAD+ levels in the cell but severely hinders DNA damage detection and repair, an effect beneficial for cancer chemotherapeutic procedures and for some muscle disorders [16,75,76].

The role of NAD+ as a substrate for CD38 and CD157 in skeletal muscle is not fully understood. The significance of this pathway is to produce a small amount (3%) of cADPr, a potent regulator of Ca^2+^ concentration [3]. In addition, other NAD+ metabolites, such as O-acetyl-ADPr, ADPr, and nicotinic acid adenine dinucleotide (NAADP) are also involved in promoting Ca^2+^ influx into muscle fibers [63,77,78]. Given the high requirement of Ca^2+^ during muscle contraction, the importance of this pathway should not be overlooked. The low efficiency of this pathway, i.e., to hydrolyze 97 molecules of NAD+ for the generation of 3 molecules of cADPr is noteworthy. Furthermore, muscle aging upregulates CD38, a major reason for the age-related decline of NAD+ in the cell (see Section 6.3 below).

### 5.2. Additional NAD+ Functions in Muscle

Recent research indicates that in addition to metabolic functions, NAD+ is involved in a wide range of cellular activities, such as supporting extracellular matrix (ECM), muscle development, and regeneration [53]. For example, NAD+ was found in the ECM and serves as a substrate for ecto-ADP-ribosyltransferase (ART) to support ADP-ribosylation of certain proteins, such as integrin and laminin, which play an important role in maintaining myofibrils in an organized manner, especially during the development of myotome [79]. NAD+ was also shown to “leak” out of cell membrane and localize to the Golgi, endoplasmic reticulum, peroxisomes, and lysosomes [53]. Adequate NAD+ concentration is essential for the adhesion of laminin to sarcolemma and the construction of the extracellular microenvironment as a whole. The exact implications of these findings are still under investigation.

Recent research suggests that NAD+ is an important co-factor for mitochondrial ADP-ribosylation. The work of Hopp et al. [80] showed that inhibition of ETC activity could increase mitochondrial protein ADP-ribosylation, whereas H_2_O_2_-induced oxidative stress decreased mitochondrial ADP-ribosylation while promoting nuclear ADP-ribosylation. Interestingly, elevated mitochondrial ADP-ribosylation attenuates H_2_O_2_-induced nuclear ADP-ribosylation, thus increasing MMS-induced ARTD1 chromatin retention. These findings support a view that NAD+ via ADP-ribosylation may play a role in the mitochondrial-nuclear crosstalk to regulate gene expression under oxidative stress.

There is evidence that NAD+ may be a nutrient sensor during muscle development [81]. Because NADH is the primary reducing power generated during intermediary metabolism, its level represents the nutritional status in the cell. When myoblasts differentiate into myotubes, there is a decrease in NAD+ levels, indicating NAD+ may be consumed [81]. Decreased NAD+ due to glucose starvation may activate AMPK, which in turn activates NAMPT activity and restores NAD+ levels [82]. Maintenance of NAD+ concentration and thus proper SIRT1 activity is important for differentiating myoblast to keep ATP production for growth while keeping differentiation at the right pace. Interestingly, the dependence of NAD+ by differentiating muscle cells only occurs during secondary (fetal) myogenesis but not during primary (embryonic) myogenesis [53]. A recent study by Sincennes et al. [83] raised the possibility that transcription factor PAX7 acetylation status may regulate muscle stem cell function and differentiation potential during the development of muscle fibers. PAX7 contains two acetylation sites that positively regulate its transcriptional activity. Hypoacetylation of PAX7 attenuates its DNA binding, whereas NAD+-induced SIRT2 deacetylates and promotes PAX7 nuclear binding. Abolishing acetylation of PAX7 promotes an expansion of the satellite cells, restricts asymmetric stem cell divisions, and overexpresses oxidative IIA myofibers. Thus, NAD+ by sustaining SIRT2 activity may play a critical role in positively regulating the expression of target genes via PAX7.

Muscle repair and regeneration depend on a successful transition from quiescent satellite cells (SC) to develop into mature cells. SC uses mitochondrial oxidative phosphorylation to generate ATP, which also seems to involve NAD+ as a sensor. Proper NAD+ levels maintain SIRT1 activity, which represses myogenic genes and keeps the SC in the quiescent state. When SC are activated and proliferating, they switch energy production to glycolysis [84], resulting in decreased NAD+ levels and, subsequently, suppression of SIRT1 activity to “permit” SC to differentiate into mature muscle cells. While lower SIRT1 activity is desirable in SC differentiation, maintenance of proper SIRT1 expression has been shown to be crucial in muscle recovery from injury in aged animals. Myers et al. [85] demonstrated that contraction force was greater after recovery from cardiotoxin-induced injury in older mice overexpressing SIRT1 than their SIRT1 KO counterparts. SIRT1 and P53 overexpression also increased fatigue resistance during repeated contraction experiments compared to WT or SIRT1 KO mice. Interestingly, a proliferation of SC was greater in SIRT1 KO older mice; however, ablation of SIRT1 in SC by gene KO significantly attenuated the ability of muscle to recover from injury. Under other stress conditions, such as a large dose of tumor necrosis factor TNF-α in vitro, SIRT1 expression was shown to be crucial in resisting apoptosis and sustaining cell survival [86]. Almada et al. [87] recently demonstrated that FBJ osteosarcoma oncogene (Fos) mRNA and protein play an important role in activating SC induced by muscle damage. Interestingly, NAD+-consuming mono-ADP-ribosyltransferase 1 (ART1) is one of these Fos/AP-1 targets, and disruption of its signaling in SC can impede progenitor cell expansion and delay muscle regeneration.

Taken together, it is clear that there exists a competitive relationship among SIRTs, PARP-1, and CD38, as they all use NAD+ as a substrate. Since PARP-1 and SIRT1 have similar Km values for NAD+ (50–97 μM for PARP-1 vs. 94–96 μM for SIRT1), a reduction in NAD+ caused by PARP-1 activation could lead to a decreased SIRT1 activity [66,88]. As mentioned before, the inactivation of SIRT1 has major implications for metabolic attenuation. Besides competing for NAD+, SIRT1 usually inhibits PARP-1 through deacetylation, thus controlling its expression [2]. Decreased SIRT1, due to a shortage of NAD+, can lead to higher PARP-1 levels, forming a negative loop to reduce NAD+ levels further. Moreover, decreased SIRT1 allows p65/Rel A to remain acetylated and activated, whereas this effect is exaggerated by transcriptional activation of NFκB by PARP-1 [89,90].

## 6. Aging Causes NAD+ Deficiency in Muscle

### 6.1. Muscle NAD+ Level Decreases with Aging

The discovery that aging could cause declines in cellular NAD+ levels came from the observation that SIRT activities were lowered in aged animals [28,38]. It was noticed that transgenic mice overexpressing SIRT1 stimulated insulin secretion and ameliorated glucose metabolism when they were young, but the positive effects faded when mice grew older. Subsequent research revealed that SIRT1 activity was compromised because of diminished NAD+, the mandatory substrate for SIRTs [38]. Supplementation of NAD+ precursor NMN was able to restore NAD+ due to the conversion of NMN to NAD+ by NMNAT and ameliorated the aging effect. Since then, a number of studies confirmed that NAD+ levels in mammals, as well as in several species of invertebrates, decrease with advanced age in tissues and organs such as the liver, brain, and muscle [28,39,91,92,93]. Skeletal muscle is one of the organs dramatically demonstrating this phenomenon [17,18,32,94,95].

In living cells, NAD+ is much more abundant than NADH [2], presumably because the latter is readily converted back to NAD+ by the ETC under normal physiological conditions. Thus, most studies use the NAD+ level as an indication of the total cellular nicotinamide adenine dinucleotide pool. Although cellular NAD+ levels have been shown to decline as much as 80% at old age [91,96], the extent of NAD+ change in skeletal muscle is less clear and variable data have been reported in the literature. Gomes et al. [28] showed that NAD+ content in the gastrocnemius muscle of 6-month-old mice was ~230 pmol/mg protein. Frederic et al. [18] showed that NAD+ concentrations in the mixed hindlimb muscles (quadriceps, gastrocnemius, and tibialis anterior) of both male and female mice were about 500 pmol/mg muscle. It is unclear whether wet or dry muscle weight was used to normalize NAD+ concentration. The authors also showed that NAD+ concentration in isolated muscle mitochondria was in several nmole ranges per mg protein. In a recent study, Yeo et al. [12] measured NAD+ concentration at 250 pmol/mg protein in mouse gastrocnemius and quadriceps muscles, similar to that of Gomes et al. [28]. Since NAD+ concentration is highly compartmented in the cell, it is difficult to know the extent of age-related NAD+ deficit in muscle. Yeo et al. [12] found that NAD+ concentration in the homogenate of mouse quadriceps and gastrocnemius muscle decreased about 50%, comparing 12 months and 24 months of age with 6 months of age. The nuclear NAD+ level was also decreased by approximately half, whereas cytoplasmic NAD+ showed only a modest decrease of ~20%. Because nuclear poles are large enough for NAD+ to exit into the cytosol, the exact concentration of NAD+ in cytosol is often uncertain. Since skeletal muscle is highly heterogeneous in fiber type compositions with different genotypic sources of originality and metabolic profile, the NAD+ level is likely to be different in different fiber types [97]. Furthermore, different fiber types age at different rates, giving even greater diversity of biochemical and physiological endowments [98]. Currently, there is a paucity of data regarding fiber-specific alteration of NAD+ turnover during aging.

### 6.2. Consequences of NAD+ Deficit in Aging Muscle

A hallmark of aging is decreased mitochondrial function, demonstrated by a reduced ability to utilize metabolic fuels, decreased ATP production, and oxygen consumption [99,100]. Mitochondrial volume also decreases in aged muscle, largely explained by decreased mitochondrial biogenesis controlled by the SIRT/PGC-1α/Tfam axis [101]. However, the mechanism of age-related decline in mitochondrial quality and quantity is not entirely clear. The discovery that aged organisms, including muscle suffer from diminished NAD+ pools, has provided a new insight to this decade-old puzzle in age research.

The enzyme that is most affected by a diminished NAD+ level with aging is SIRTs, especially SIRT1 and SIRT3. SIRT activity systematically declines in aging despite the relatively stable enzyme protein content [1]. This decline can directly affect its ability to deacetylate PGC-1α, leading to a lower Tfam, the primary nuclear factor to activate mitochondrial biogenesis [2]. SIRT1 is also known to activate mitochondrial enzyme expressions via a PGC-1α-independent, but HIF-dependent pathway [18]. In addition, decreased NAD+ also inactivates SIRT3, the mitochondrial sirtuin, thus causing inhibition of enzymes in the TCA cycle through increased acetylation [102]. Moreover, lower mitochondrial NAD+ could hinder the ability of Complex I to oxidize NADH and restrict electron flow through the ETC [67,69,70,103]. Thus, a compromised NAD+ level may provide a direct explanation of the age-related loss of mitochondrial homeostasis, which leads to muscle functional loss and sarcopenia. Indeed, in a recent study, Yeo et al. [12] demonstrated that NAD+ deficit in 24-month-old mice was associated with increased acetylation of a wide range of proteins, such as PGC-1α, GCN5, p65, and SOD2 in mouse hindlimb muscles and heart. Ironically, protein levels of SIRT1, 3, 5, and 6 were upregulated comparing old vs. young muscles. Other authors have also reported decays of SIRT activities with aging, and directly attributed SIRT downregulation to NAD+ deficit [28,38,104]. These data emphasized the importance of cellular NAD+ in controlling overall acetylation status and thus, muscle functionality.

Another serious consequence of decreased NAD+ availability in an aged organism is compromised antioxidant defense, leading to increased oxidative stress and inflammation, termed “inflammaging” [3]. First, aged muscle is known to generate high levels of ROS primarily in the mitochondria, but also due to inflammation [101,105]. Diminished NAD+ levels and SIRT3 activity can attenuate SOD2 deacetylation and thus, the ability of removing superoxide anion. Acetylation increases the binding activity of p65, a crucial step in the transactivation of pro-inflammatory cytokine expression [61,62]. Moreover, lowered NAD+ level limits the ability of PARP-1 to utilize pADPr to repair DNA damage that occurs at old age [106,107].

### 6.3. Potential Mechanisms of Age-Related NAD+ Decline

Cellular mechanisms for decreased NAD+ levels with aging are multifaceted, related to both NAD+ consumption and synthesis. The most important reason is probably related to increased protein acetylation in aging organisms [1,3]. Proteomic analysis reveals that over 100 lysine sites in mitochondrial proteins are acetylated, which can be one of the most common post-translational modifications of mitochondrial homeostasis [108]. Aging is a prominent inducer of mitochondrial protein hyperacetylation, the major cause of mitochondrial enzyme dysfunction in the TCA cycle and ETC, loss of redox homeostasis, and increased organelle oxidative damage [11,55]. A recent study revealed that hindlimb muscles of 24-month-old mice had enhanced acetylation of PGC-1α by 6-fold, p65 by 4-fold, SOD2 level by 8-fold, GCN5 by 50%, and total protein level by 3-fold, compared to those of 6-month-old counterparts [12]. Importantly, most of these parameters showed significant elevations at 12 months of age, indicating age-dependent NAD+ deficit might start earlier than previously thought. Increased protein acetylation requires higher SIRT activity for deacetylation, and in turn, more NAD+ to accept acetyl moieties from acetylated proteins [1,109]. Whether aging upregulates or downregulates SIRT1 is still controversial. The ironic finding was that despite increased protein expression of SIRT1 in response to aging, its activity is unchanged or even decreased due to diminished NAD+ supply in muscle cells [94,110].

Besides increased consumption by SIRTs, aging has been shown to increase CD38 and CD157 activities [34]. Cleavage of NAD+ generates ADP-ribose and NAM, used as DNA damage repair and for NAD+ salvage, respectively. Recent studies reported that aging gradually increases CD38 protein levels and its NADase activity [17,32,33,35]. CD38 gene knockout and 78c, a specific CD38 inhibitor, rescued intracellular NAD+ and preserved SIRTs activity [17,32]. It was demonstrated that CD38 expression increased by 2–6-fold in the skeletal muscle of mid-aged mice and by 5–13-fold in the old mice, supporting the view that upregulation of this enzyme, could be the main reason for muscle NAD+ deficit at old aged [12,111,112].

Another explanation for age-associated decreases in muscleNAD+ is an upregulation of PARP-1, which uses NAD + as a substrate to catalyze the covalent transfer of ADP-ribose for DNA repair [1]. Elevated PARP-1 levels may be an inevitable process of aging due to an accumulation of DNA damage [106]. Treatment of PARP-1 inhibitor was shown to increase NAD+ pools and elevate SIRT activity [9,106,113]. Skeletal muscles of old mice accumulated higher levels of cleaved pADPr, suggesting that PARP-1 activity was increased during aging [12]. Taken together, aged muscles clearly suffer from a NAD+ deficit, which might be attributed to the enhanced deacetylation demand catalyzed by SIRTs and the upregulation of two enzymes that consume NAD+, namely CD38, and PARP-1.

While clear evidence exists that aging increases NAD+ degradation in muscle, research also indicates that NAD+ synthesis may diminish at old age. As previously mentioned, the primary means to replenish cellular NAD+ is the NAD+ salvage pathway catalyzed by its rate-limiting enzyme NAMPT. There is a consensus that aging decreases NAMPT expression in several tissues, including skeletal muscle [2,114]. Age-associated downregulation of NAMPT is probably caused by a defective circadian rhythm regulation by CLOCK and BMAL [39,115]. BMAL and CLOCK control the expression of NAMPT, whereas they are regulated by SIRT1 through the deacetylation of a central clock component in the liver [116,117]. An age-related decline in SIRT1 activity may be the primary reason for the observed downregulation of NAMPT.

NAMPT downregulation during aging may also be triggered by increased inflammation, marked by elevated pro-inflammatory cytokine expression. TNF-α inhibits BMAL/CLOCK-mediated transcription in hepatocytes and hence, NAMPT expression [39,118]. Since aging is associated with increased muscle inflammation, marked by elevated TNF-α and other inflammatory triggers, such as cyclooxygenase 2 (COX2), suppression of muscle inflammation may provide an alternative strategy to maintain proper NAD+ and SIRT levels during aging [119].

### 6.4. Supplementation of NAD+ Precursors Ameliorates Muscle Aging 

At a young age and in a healthy state, NAD+ levels do not seem to limit cardiac and skeletal muscle physiology [120]. However, decreases in NAD+ content are apparent in the brain, liver, and muscle, coincident with functional declines in these tissues at older age [9,28,91,93]. Furthermore, increased NAD+ levels are associated with increased longevity in the invertebrates and improve metabolic function in rodents [2,3]. Therefore, various strategies have been postulated and experimented aiming to restore and boost cellular NAD+ pools, making NAD+ supplementation a hot area of both basic research and human clinical trials. It is noticed that supplementation of four different NAD+ precursors has demonstrated age-specific efficacy not necessarily in agreement with the observation of younger animals or humans.

Since mitochondrial dysfunction is considered the hallmark of muscle aging, the majority of research has linked NAD+ precursor supplementation to mitochondrial function. It is generally agreed that the primary reason for NR supplementation to restore NAD+ levels and ameliorate aging effects in muscle is due to increased SIRT1 activity, thus activating the SIRT1/PGC-1α/Tfam axis and improving mitochondrial homeostasis [42,121,122,123]. NAMPT overexpression alone augmented endurance performance in mice, demonstrating the efficacy of the salvage pathway [124]. While NAM supplementation alone may not be effective in raising cellular NAD+ levels, physical exercise in conjunction with NAM has shown some promise. For example, Pajk et al. [125] supplemented NAM in drinking water to young and old mice in conjunction with endurance training. NAM treatment increased SIRT1 deacetylation activity in both age groups, accompanied by an increased PGC-1α level in older mouse muscle. These data provided some promise that oral NAM supplementation, especially combined with exercise, may not cause SIRT1 inhibition in vivo. Recently, Das et al. [126] demonstrated that supplementation of NAM increased blood flow and endurance capacity in old mice by promoting SIRT1-mediated capillary density. The authors emphasized the role of blood flow in aging muscle and highlighted the role of SIRT1 in angiogenesis to elevate hydrogen sulfide (H_2_S) and restore NAD+ levels in the endothelial cells. Long-term administration of NMN was found to be an effective regimen to mitigate an age-associated physiological decline in mice [127]. Twelve months of NMN supplementation with a regular chow diet in C57BL/6N mice suppressed body weight gain, enhanced energy metabolism, promoted physical activity, and improved insulin sensitivity during normal aging. Thus, NMN may be a more direct and simpler dietary source to ameliorate aging effects.

Another potential site of action for NAD+ precursors to ameliorate muscle aging is muscle stem cells (MuSC), as revealed in a study by Zhang et al. [128]. NAD+ concentration in MuSC from aged mice was lower than that from young mice, indicating NAD+ deficit was the main reason for these mice demonstrating a range of muscle dysfunctions, whereas supplementation of NR increased NAD+ levels in MuSC. NR rejuvenated age-associated MuSC regeneration, mitochondrial function, and SIRT1 activity. Importantly, NR also increased muscle strength, running duration, and life span in aged mice [128]. The study shed new light to the role of NAD+ in improving not only the existing senescent myocyte function but also the ability to reverse muscle morphological and physiological declines at old age via stem cell rejuvenation.

Due to the importance of the salvage pathway in recycling NAM to synthesize NAD+, the role of NAMPT as the rate-limiting enzyme for this pathway, and as a key factor for ameliorating aging, was highlighted in a recent review [51]. Transgenic overexpression of NAMPT has been reported to improve cellular functions and attenuate the onset of senescence in several tissues, including skeletal muscle [120]. NAD+ levels were shown to increase by 2-fold in transgenic lines of mouse embryotic fibroblast (MEF) compared to WT MEF cells [51]. Increased SIRT1 activity, elevated antioxidant enzyme expression, and resistance to oxidative stress were also observed in this cell line. The authors also explored several small molecular weight pharmacological compounds as potential activators for NAMPT in vitro and in vivo. However, none of them was shown to be effective in boosting muscle NAD+ levels [51].

It is noteworthy that NAD+ precursor supplementation is still a controversial subject and the research outcome of its efficacy, especially in humans, is mixed. Several recent clinical trials revealed that chronic NR supplementation did not improve mitochondrial volume and function in humans [44,46,47,129], even though NAM and nicotinic acid riboside (NAR) contents were elevated [44]. These studies challenged the proposition that aged people should increase consumption of vitamin B3 compounds for the sake of preserving muscle function. For example, Connell et al. [130] failed to observe increases in NAD+ levels or limb muscle functional improvement conducted by MRI scanning after one-month supplementation of a mixture of niacin equivalent (tryptophan, NA, and NAM). Noticeably, NR was missing from the ascribed dietary source. Stocks et al. [44] also did not observe a significant change in muscle NAD+ content or mitochondrial functional capacity following 7-days of NR supplementation. Clearly, the efficacy and mechanism of NR supplementation in ameliorating functional improvement of aging muscle require more innovative and thorough investigation, and the effects of exercise in ameliorating dietary supplementation seem to be critically important [37].

## 7. Exercise Adaptation of NAD+ Homeostasis

Skeletal muscle is an organ that demonstrates a wide range of functional plasticity through dynamic metabolic adaptations to energy needs, fuel diversity, and redox changes. Due to the multifaceted roles of NAD+ in transferring reducing power (via NADH), supporting mitochondrial biogenesis and function (via the Sirt1/PGC-1α axis), maintaining Ca^2+^ homeostasis (via CD38), and repairing DNA oxidative damage (via PARP-1), it is expected that NAD+ levels may reveal more dynamic perturbation during muscular contraction, especially under stressful conditions. Furthermore, skeletal muscles are heterogeneous entities with different percentages of type I and II fibers, and as a result, morphological properties, such as MyHC expression and fiber size, and metabolic profiles, such as oxidative enzyme levels and blood supply, also differ in response to aging and exercise [97,98]. Indeed, new research has emerged showing that both acute and chronic exercise can significantly alter NAD+ homeostasis in skeletal muscle. Furthermore, exercise may exert more dramatic effects on aged muscles, which already are under various challenges due to nutritional variability, oxidative stress, and declined habitual activity [131].

### 7.1. Response of NAD+ to Acute Exercise

NAD+ is required for a wide range of metabolic pathways that are activated during an acute bout of exercise. When the fuel of muscle contraction is primarily from carbohydrates during high-intensity exercise, NAD+ is converted to NADH when the glycolytic intermediate glyceraldehyde 3-phosphate is oxidized to 3-phosphoglyceryl phosphate by GAPDH. Due to low cellular oxygen levels and slow rate of mitochondrial oxidative phosphorylation, NADH transportation into the mitochondria and oxidation by Complex I (NADH reductase) of ETC is low. Therefore, NADH reacts with pyruvate to form lactate, subsequently restoring the cytosolic NAD+ pool to allow for continuous glycolytic flux. Muscle NAD+ and NAD+/NADH ratios depend on exercise intensity, and the response also seems to be different between animals and humans and between muscle fiber types [20]. Furthermore, most attention in previous studies seems to be on NADH instead of NAD+ levels. At exercise intensity greater than 75% VO_2_max, NAD+ levels in human muscle show no significant changes or even an increase [132]. After exercise, at 75% and 100% VO_2_max, muscle NAD+ levels, and the NAD+/NADH ratio increased above resting values in both types I and II fibers, while type I fibers showed higher NADH content than type II fibers [20,133]. The increase of NADH in both fiber types at high-intensity exercise suggests that the availability of oxygen relative to the demand is decreased. However, during submaximal exercise at 50% VO_2_max, there is a decrease of total muscle NADH concentrations due to elevated mitochondrial ETC activity [134]. Moreover, after exercise at 40% VO_2_max, muscle NADH decreased primarily in type I fibers, whereas no significant change in NADH was observed in type II fibers [133]. During an acute bout of aerobic exercise, NAD+ is a substrate for GAPDH and pyruvate dehydrogenase (PDH) to form NADH. Cytosolic NADH is transported into the mitochondrion by the malate-aspartate shuttle or glycerophosphate shuttle, joining the pool of NADH produced by ICDH, α-KGDH, and MDH. Increased ETC activity increases NADH oxidation and thus NAD+/NADH ratios within the organelle, which is separated from the cytosolic NAD+ pool [2,3]. Since the majority of nicotinamide dinucleotide pool is in the form of NAD+, the classic view is that the changes in NADH and NADH/NAD ratio do not substantially impact NAD+ reserves, as long as exercise intensity does not exceed 75% VO_2_max [134,135].

However, the classic view of the NAD+ dynamics was reconsidered dramatically during the past several decades due to the discoveries that the various pathways that determine cellular NAD+ content, as discussed in previous sections, can be influenced significantly by exercise. Specifically, exercise may alter NAD+ levels not only due to increased consumption by SIRT1 and PARP-1 but also due to altered flux of the NAD+ salvage pathway.

In a landmark paper, Gerhart-Hines et al. [66] demonstrated that SIRT1 is a required enzyme to sense the low glucose concentration and activate PGC-1α to boost mitochondrial biogenesis and fatty acid metabolism. They found that the inclusion of NAM, an NAD+ precursor, could modulate SIRT1 response and thus postulated that SIRT1 activity might be regulated by NAD+ availability. In a previous study by the same group, they showed that deacetylation of PGC-1α by SIRT1 was NAD+-dependent and that increased NAD+ levels correlated with PGC-1α deacetylation [136]. It is important to note that PKA can phosphorylate SIRT1 and increase SIRT1 catalytic activity by lowering its Km for NAD+ [42]. Thus, at the same cellular NAD+ concentration, SIRT1 activity may be elevated during acute exercise due to the shifted enzyme kinetics. This altered kinetic profile may also decrease NAD+ consumption by SIRT1, making it function more efficiently. Canto et al. [137] reported that an acute bout of swimming increased deacetylation of PGC-1α and FoxO1 in mouse skeletal muscle and increased NAD+ levels, which coincided with elevated NAMPT mRNA abundance. Interestingly, these effects of exercise were absent in AMPK KO mice, suggesting AMPK acts as the sensor that transmits the signal to SIRT1. This finding was confirmed by Brandauer et al. [138] demonstrating that NAMPT mRNA expression in leg muscle was elevated 3 h after an acute bout of treadmill running in the wild-type mice, but not in AMPKα2 KO mice [138].

Heavy exercise is known to cause DNA oxidative damage in skeletal muscle marked by increased 8-OH-dG levels [25]. Increased oxidative modification of DNA requires not only 8-oxoguanine glycosylase (OGG1) to cleave the glycosidic bond and break damaged DNA base pairs, but also PARP-1 to transfer pADPr to repair damaged DNA strand, resulting in increased consumption of NAD+ [94]. However, the effect of exercise on PARP-1 has been reported sparsely. Mohamod et al. [75] showed that muscle contraction with electric stimulation increased ADP-ribose production as evidence of PARP-1 activation, whereas PARP-1 mRNA expression was elevated. A study by Rajmohan et al. [90] clearly demonstrated that there is a crosstalk between SIRT1 and PARP-1. When cardiomyocytes were mechanically stressed, PARP-1 was acetylated and activated. SIRT1 physically binds to and deacetylates PARP-1 in vivo. The authors also reported an age-related decrease in PARP-1 and SIRT1 activities, as well as lower NAD+ levels in older mice. These data indicate that contraction-mediated PARP-1 activation may play a role in reducing NAD+ homeostasis.

The overwhelming evidence that NAD+ deficiency impairs skeletal muscle function and that NAD+ consumption is increased during acute exercise, as discussed above, implies that supplementation of NAD+ or NAD+ precursors may ameliorate multiple pathways supported by NAD+ and thus improve muscle performance. However, few studies, if any, have reported such an “ergogenic” effect of NAD+ supplementation. Kourtzidis et al. [139] administered NR to rats at a dose of 300 mg/kg body wt/day for 21 days via gavage, followed by an endurance test. Surprisingly, NR-supplementation resulted in a 30% reduction in endurance time. A follow-up study by the same group of investigators using the same dietary regimen revealed no benefits of NR supplementation, but attenuated erythrocyte antioxidant enzyme activity, increased blood oxidative stress, and a tendency toward anaerobic metabolism during exercise [139]. These data confirmed a general conclusion that in a young and healthy state, metabolic function and probably muscle performance are not limited by NAD+ levels in the cell [1,2]. Noticeably, several studies supplementing NAM in aged mice reported improved endurance capacity in association with increased capillary blood flow [125,126]. Interestingly, NAMPT overexpression alone was able to augment endurance performance in mice, demonstrating the potential link between NAD+ salvage and metabolic function in muscle [124].

### 7.2. Adaptation of NAD+ to Chronic Exercise

In contrast to the effect of an acute exercise bout, chronic exercise appears to increase cellular NAD+ levels, and this view has been supported by both animal and human studies. Koltai et al. [94] showed that exercise training increased NAD+ levels in the skeletal muscle of young rats and mitigated NAD+ deficit in muscle of aged rats. The exercise adaptation was associated with a training induction of NAMPT1 in both young and older muscles. The authors also demonstrated higher SIRT1 activity and lower global protein acetylation after training, despite lower SIRT1 protein content with aging. They attributed this effect to higher NAD+ content found in trained muscle. Uddin et al. [140] reported that endurance training by treadmill running for 6 weeks increased NAD+ content in skeletal muscle and liver of mice injected with NMN (0.5 g/kg body wt) during the last 17 days of training. The training effect was not observed in mice injected with PBS buffer.

The mechanism for ameliorated NAD+ homeostasis with endurance exercise is likely multifaceted, but most research seems to point to the induction of NAMPT, the rate-limiting enzyme in NAD+ salvage pathways [37]. NAMPT is ubiquitously expressed in various tissues of animals, including skeletal muscle [141]. Embryonic knockout of NAMPT in mice is lethal [142,143]. Exercise training has been identified as a potent stimulus to increase NAMPT levels in skeletal muscle [124,138,144]. Additionally, skeletal muscle NAMPT protein levels are higher in endurance-trained athletes compared to untrained individuals [145]. De Guia et al. [146] recently showed that 12 weeks of aerobic training increased NAMPT levels by 12% and 28% in young and older individuals, respectively, while NAMPT abundance in skeletal muscle correlated highly and negatively with age. Moreover, resistance training increased NAMPT abundance by 25% and 30% in young and in older individuals, respectively. Lamb et al. [147] reported that while muscle NAD+ and NADH levels were decreased in the vastus lateralis muscle of middle-aged men, NAD+ and NADH abundance and SIRT activity were elevated two-fold after training, along with modest increases in NAMPT protein content and SIRT1 activity.

Endurance training in rodents and humans increases SIRT1 and SIRT3 protein levels, as well as deacetylation activity in skeletal muscle [94,148,149]. The adaptation may facilitate deacetylation and activation of NAMPT [75]. In addition, NAMPT expression in skeletal muscle is regulated, at least in part, by AMPK, as exercise-induced NAMPT mRNA abundance was abolished in AMPK KO mice [137,138]. In the mouse, NAMPT training adaptation was dependent on AMPKα2 but independent of PGC-1α [138].

Based on the above-mentioned studies, endurance exercise seems to be capable of maintaining and even elevating muscle NAD+ levels, mainly due to the activation of NAMPT in recycling NAM produced by SIRTs and PARP-1. However, an alternative pathway for NAD+ synthesis bypassing NAMPT has received considerable attention recently. This pathway uses dietary NR to generate NMN catalyzed by the muscle-specific NRK2 [10,43]. NMN can be converted to NAD+ with a single step catalyzed by NMNAT, an enzyme playing a dual role in the Preiss–Handler pathway and the salvage pathway [2]. NR has demonstrated high bioavailability and safety in clinical trials and has received the GRAS (Generally Recognized as Safe) status by the FDA [150]. Notably, NR appears more effective in preserving muscular functions in aged animals and in humans [128,151], whereas *Nmrk2*^−/−^ mice failed to increase NAD+ levels in skeletal muscles in response to endurance training [152]. Deloux et al. [153] demonstrated that 9 weeks of treadmill training elicited no effect on muscle NAD+ levels in *Nmrk2*^−/−^ mice. Training-induced muscle morphological adaptations were largely missing in this phenotype, suggesting NRK has a special role in regulating NAD+ homeostasis and muscle function. Recently, Crisol et al. [154] studied the effect of NR supplementation in combination with exercise training. NR alone did not change animals’ performance, but NR increased muscle NAD+ levels after training. NMNAT3 protein levels positively correlated with several mitochondrial functional markers, and combining NR supplementation and training increased aerobic performance compared to training per se, and promoted the proportion of type I fibers in muscle.

## 8. Conclusions

The recognition of NAD+ as a versatile multifunctional molecule was made mostly during the past two decades. As a coenzyme, NAD+ is the major reducing power supporting numerous enzymes in transporting hydrogen ions in metabolic pathways. As a signaling molecule, NAD+ serves as the substrate for SIRTs, PARP, and CD38/CD157 and plays a crucial role in protein deacetylation, providing pADPr to repair DNA, and producing a second messenger for Ca^2+^ regulation. During these processes, cellular NAD+ levels diminish and require constant replenishment. The primary means to restore NAD+ pools is the salvage pathway, during which NAM is combined with the relatively abundant ATP and ribose molecules to regenerate NAD+ by NAMPT. It seems that young and healthy organisms can reach NAD+ homeostasis with the above mechanisms.

Physical exercise increases metabolic and oxidative stress to skeletal muscle requiring higher NAD+ turnover. Exercise increases NAD+ consumption by SIRTs to deacetylate critical enzymes and transcription factors, and by PARP-1 to repair the DNA polynucleotide chain. This demand seems to be met mostly by the upregulation of NAMPT to recycle NAM, but the efficiency of NAD+ restoration may be limited by cellular sources of NAD+ precursors.

**Figure 2:**
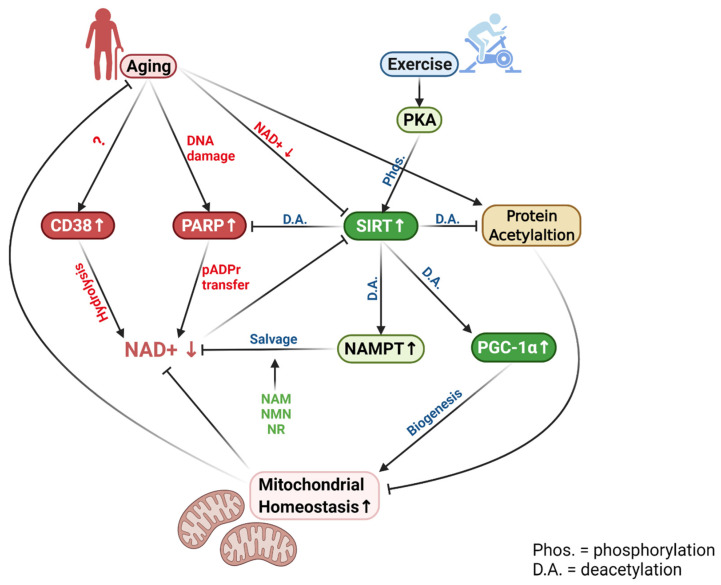
The effect of aging and exercise on NAD+ homeostatic regulation in muscle. Aging increases the demand on NAD+ due to the activation of SIRTs, PARP-1, and CD38. Decreased NAD+ content reduces SIRT1 activity, which plays a key role in mitigating protein acetylation and maintaining mitochondrial homeostasis during aging. NAD+ levels may be partially restored by higher NAMPT activity and by supplementation of NAD+ precursors. For abbreviations, see the list at the end of the paper. Figure created with BioRender.com.

Aging gradually tilts the balance between NAD+ consumption and biosynthesis in favor of the former. Increased acetylation of proteins, enzymes, and key transcription factors in the cell demands a higher SIRT activity, while PARR-1-catalyzed pADPr cleavage and CD38-catalyzed NAD+ hydrolysis are increased with aging, resulting in a net NAD+ deficit. Dietary supplementation of NAD+ precursors, such as NA, NMN, and NR can utilize the existing enzymatic pathways to replenish cellular NAD+ pools and thus, ameliorate SIRT functions to stimulate mitochondrial homeostasis without sacrificing PARP-1 activity to restore the integrity of DNA and other vital proteins during aging (Figure 2).

## Abbreviations

ACMS	2-amino-3 carboxymuconate semialdehyde
ART	ecto-ADP-ribosyltransferase
BCKADH	α-ketoacid dehydrogenase
COX2	cyclooxygenase 2
FAS	fatty acid synthase complex
GAPDH	glyceraldehyde phosphate dehydrogenase
GDH	glutamate dehydrogenase
GPDH	glycerol-3 phosphate dehydrogenase
GR	glutathione reductase
HADH	3-hydroxylacyl coenzyme A dehydrogenase
ICDH	isocitrate dehydrogenase
α-KGDH	α-ketoglutarate dehydrogenase
LDH	Lactate dehydrogenase
MDH	malate dehydrogenase
NA	nicotinic acid
NAAD	nicotinic acid adenine dinucleotide
NAADP	nicotinic acid adenine dinucleotide
NADS	NAD+ synthase
NAMN	nicotinic acid mononucleotide
NAMPT	nicotinamide phosphoribosyltransferase
NAR	nicotinic acid riboside
NMN	nicotinamide mononucleotide
NMNAT	NAMN transferase
NR	nicotinamide ribose
NRK	nicotinamide ribose kinase
OGG1	8-oxoguanine glycosylase
pADPr	poly ADP-ribose
PARP	poly (ADP-ribose) polymerase
PDH	pyruvate dehydrogenase
QA	quinolinic acid
QAPRT	quinolinic acid phosphoribosyltransferase
SIRTs	sirtuins
TR	thioredoxin reductase
